# Improving the design of a mCSCL Chinese character forming game with a distributed scaffolding design framework

**DOI:** 10.1186/s41039-017-0066-4

**Published:** 2017-12-29

**Authors:** Lung-Hsiang Wong, Chee-Kit Looi, Ivica Boticki

**Affiliations:** 1 National Institute of Education, Nanyang Technological University, Singapore, Singapore; 2 University of Zagreb, Zagreb, Croatia

**Keywords:** Distributed scaffolding design framework, Mobile-assisted language learning (MALL), Mobile computer-supported collaborative learning (mCSCL), Design-based research

## Abstract

Appropriate design of collaborative learning activities for students using mobile devices can be supported by different forms of scaffolding provided by peers, by the teacher or by the technology. Building on prior studies in mCSCL (mobile computer-supported collaborative learning), we developed Chinese-PP, a novel in-class mobile synchronous collaborative learning game for constructing Chinese characters from components, with the unique characteristic of spontaneous small group formations. In this paper, we propose a distributed scaffolding design framework to guide us in examining and refining/revising the interplay among various forms of scaffolding in the learning model across various design-based research (DBR) cycles of our study on Chinese-PP in a primary school in Singapore. We believe a generalized scaffolding design framework has the potential to inform technology-enhanced learning research with a structure to support the iterative process of enacting and redesigning the socio-techno-pedagogical frameworks developed by individual research projects.

## Introduction

One-to-one (1:1, one-mobile-device-per-student) technology-enhanced learning (TEL) environments transform classroom dynamics as individual students can carry mobile devices with wireless affordances into the classrooms (Liu and Kao 2007). Nevertheless, the incorporation of the 1:1 infrastructure into the classroom may impose additional challenges on the teachers in trying to cope synergistically with content, pedagogy, and technology. To go beyond the clinical stages of research and turn such designs into regular classroom modules, various aspects such as theoretical and curricular fit, pedagogical guidance, technological design, and logistic support need to be addressed (Roschelle et al. 2010).

Appropriate design of collaborative learning activities for students using 1:1 handheld devices might include elements of scaffolding provided by the technology, by peers or by the teacher that strengthen students’ collaborative skills (Boticki et al. 2011). Building on such prior studies in mCSCL (mobile computer-supported collaborative learning), we created a novel in-class mobile synchronous collaborative learning game with the unique characteristic of spontaneous small group formations. We refer to our system as “Chinese-PP,” where PP refers to 拼一拼 or “Pīn yì Pīn” in Chinese, which means “trial assembling,” or in a more colloquial meaning, “working hard.” In the game, students follow or adapt the peer scaffolds imposed both by the teacher and by the computer system. In order to complete the learning tasks, they have to draw upon their social relationships with other students to negotiate and optimize their solutions.

In this paper, we reflect on the process in which we iteratively developed and refined the scaffolding support. We present a generic framework for designing distributed scaffolding that has guided us in examining and refining (or revising) the interplay among various forms of scaffolding in the learning model across various design-based research (DBR) cycles of our study on Chinese-PP. We adopt DBR to carry out the *iterative* process of designing, experimenting, reflecting upon, and redesigning the learning model and applications, and to integrate design principles with technological affordances to render plausible solutions (Brown 1992; Collins 1992). We will narrate two completed DBR cycles of our study to demonstrate how our scaffolding design framework has assisted us in accomplishing crucial revamps in the learning and technological design, in addressing the challenges we encountered and in preserving good practices that emerged among the students and the teacher during the study.

A generalized scaffolding design framework may inform TEL research of a methodology to embark on the iterative process of improving the socio-techno-pedagogical structures developed by individual studies. In this regard, this paper is not positioned as a theoretical or an empirical paper. Instead, it fits into the field of “implementation research” (e.g., Barab and Luehmann 2003; Farrar et al. 1980; Spillane et al. 2002), which refers to the study of the methods or processes to promote the uptake of research findings, i.e., to explore the challenges that are faced when applying and adapting promising research designs to fit local or specific contexts or settings. Due to the space constraints, we will not provide details in the experimental design and data analysis (such details are available in our prior publications: Wong et al. (2011a); Wong et al. (2013)). We will instead focus on narrating our research journey in designing, revising, and refining Chinese-PP using the perspective of the design framework.

## Design-based research for learning design and re-design

DBR is a methodology commonly used by Learning Scientists. It emphasizes eventual adoption in school practices and therefore must be situated in real-life learning environments where there is no attempt to hold variable constant (Roschelle et al. 2010). Instead, design-based researchers try to optimize as much of the design as possible by observing how the different variables and elements are working out (Barab and Squire 2004). With such a methodology, the invention-revision cycles are iteratively conducted. Thus, conjectures are generated and perhaps refuted; new conjectures are developed in the next cycle and again subjected to test. The intended outcome by the end of each cycle is an explanatory framework that specifies expectations that become the focus of investigation during the next cycle of inquiry (Cobb et al. 2003). Given its nature, it is in general not suitable to employ a control group in DBR studies.

## Towards a 1:1 solution for mCSCL Chinese character learning

The Chinese-PP study is an attempt to tackle the primary hurdle of learners of Chinese as a second language (L2)—the logographic nature of the Chinese script (Fan et al. 1987; Wong et al. 2011b). Underlying the Chinese scripts is a rule-based system—each Chinese character comprises of one or more components, spatially arranged according to a limited set of orthographic rules. The traditional way of teaching Chinese characters is either through strokes or through whole characters as an image, bypassing the components. As compared to traditional ways of memorizing each character as a whole or focusing on the strokes of a character and their sequences, teaching the structure of characters and simultaneously addressing the relationship between components and wholes generates positive effects on character learning (Anderson et al. 2002; Nagy et al. 2002). This is relevant to orthographic awareness, which refers to the awareness of individual Chinese characters’ internal structures and therefore being able to infer meaning and pronunciation (Ho and Bryant 1997; Jackson et al. 2003; Shen 2005). When learners start to cultivate this orthographic awareness, which is a metalinguistic awareness, they also start the process of transforming their knowledge of characters from explicit to implicit (Jiang 2006), from performance towards the competence.

**Fig. 1:**
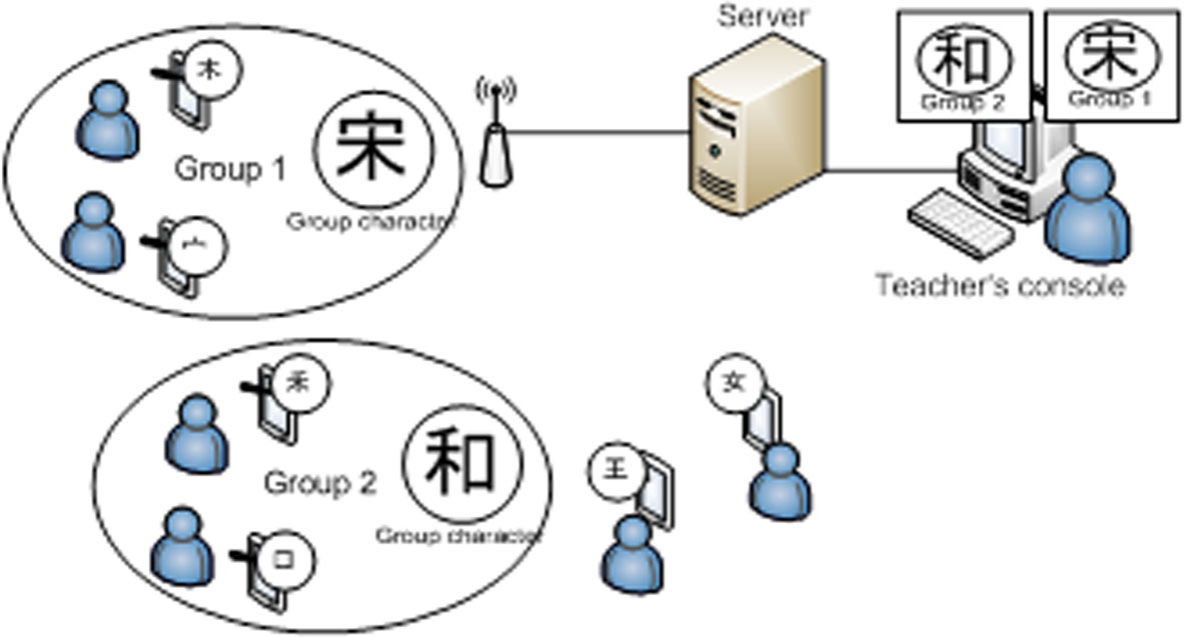
A broad architecture overview of the Chinese-PP system

Building on prior mCSCL work by Zurita and Nussbaum (2004) and our previous explorational study of a fraction addition mobile game (Boticki et al. 2011; Boticki et al. 2013), we designed the Chinese-PP character forming activity to run on 1:1 TEL setting. In Zurita and Nussbaum’s work, students were assigned to fixed small groups of three before the beginning of the classroom activities. We adapted the activity design so that students have to identify and negotiate with other students to form their own groups spontaneously with no restrictions of having fixed size to a group. In each round, a set of Chinese components is assigned by the system server via a 3G network to individual students’ smartphones. Students form groups by composing appropriate characters out of their assigned components and arrange them spatially at the correct positions to form valid Chinese characters. For example, with the components女, 口, and 月, students could place the components in the correct order to form the character 娟 (“graceful”). In addition, students are encouraged to form larger legitimate groups (groups of more than two members) for composing more complex characters. Figure 1 shows the architecture overview of the technological solution for Chinese-PP.

## The distributed scaffolding design framework

We envisaged that the Chinese-PP activity would consist of three main scaffolding sources: technological, peers, and teacher. Technology provides scaffolding in the sense of requiring the students to play the activity by following both generic and domain-specific rules and logic (in the form of software features or affordances). Peer scaffolding is encouraged in order to increase student interaction and collaboration (Boticki et al. 2011).The teacher acts as facilitator and helps the students in dealing with impasses they might encounter.

**Fig. 2:**
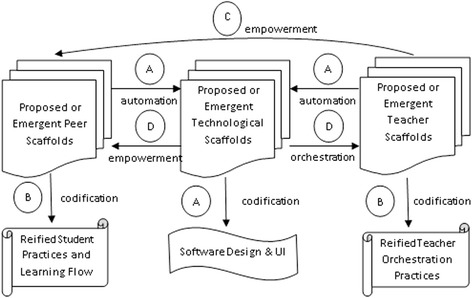
The distributed scaffolding design framework

Adopting a DBR methodology, we propose a generic framework (Fig. 2) for examining the potential roles of technological, peer, and teacher scaffolding in CSCL. The essential idea is that through the school-based experiments, different forms of peer and teacher scaffolding may emerge during the learning activities. The researchers, the teachers, and even the students can then propose new forms of scaffolding in retrospectively addressing the pitfalls that they encounter during the experiments. They could subsequently identify those which can be fully or partially automated as technological scaffolding (arrows A in Fig. 2) and distil those which should best remain in the form of teacher or peer scaffolding. The latter then becomes part of a teacher facilitation guide or student strategy guide (arrows B). Certain teacher scaffolds might be able to be replaced by or assisted with the peer help (arrow C), thus leveraging the social interactions of the class.

In addition, the scaffolding analysis can be extended to the identification of the critical success factors in learning environments and the assessment of the roles of the technological supports. For example, if teachers and students can do better in providing certain scaffolds, then the role of the technology can be revised accordingly (arrows D). Such a framework may guide us in redefining the roles of the teachers, the students, and technology through empirical research to improve the students’ learning experiences and outcomes. When good scaffolding leads to positive learning processes and outcomes, these can be reified by creating representations which can serve as best practices to help future learners and teachers. The framework is our abstraction of the design-based empirical process of designing the different forms of distributed scaffolding to work in redundant and synergistic ways (Tabak 2004).

While scaffolding is initially performed by teachers or by peers, the need for technological scaffolds can emerge and then be designed to support teacher orchestration (Dillenbourg and Jermann 2010) which is about helping teachers to manage the classroom learning activities. As the design framework informs design based on the emergent processes in real classrooms, it may present more robust and synergistic patterns of scaffolding that can withstand the in-situ interplay of various classroom constraints such as robustness of the technology, limited curriculum time, and limited energies or attention span of the students and the teacher.

What the framework advocates is a twist of the “fading” mechanism in the conventional sense of scaffolding. The teachers’ scaffolds need not be fading out but are transformed into student scaffolds. The student scaffolds may fade out by itself when individual students become more adept in the target knowledge and skills and therefore need less support from their peers.

## Chinese-PP: study description

Our initial base learning design of the mCSCL practice is informed by language acquisition theories (e.g., Bialystok 1978; Gasser 1990; Krashen 1985). To evaluate the design, we worked with our collaborating school, a neighborhood primary school in Singapore and planned two DBR cycles. The distributed scaffolding design framework has been applied across the two cycles to continuously improve the design of the software and the scaffolding support which the teacher and peers can provide.

- First cycle—formative evaluation of the base learning design: We focused on preliminary game rule design, development of technological solutions, and formative evaluations of these aspects. Two rounds of trial runs (two “micro-cycles” hereafter, known as micro-cycle 1.1 and micro-cycle 1.2 respectively) were conducted with small groups of primary 3–5 (9–11 years old) students. The students just played the game with the prescribed rules so that we could identify emergent learning patterns, iron out logistic issues, and probe the students on their perceptions of the design.
- Second cycle—pilot classroom lessons: We developed a curriculum framework to facilitate a series of learning and game playing sessions to foster students’ learning growth over the time (six sessions with 2-week intervals) of the trial. The Chinese language teacher in the primary 3 experimental class took the lead in designing and enacting individual lesson plans while the researchers played a supporting role.

In the following sections, we will describe our trials, findings, and refinements in the first and second cycles.

## First cycle, micro-cycle 1.1: the experiment and the subsequent improvement

Within the first cycle, we conducted two micro-cycles of design, evaluation, and re-design with Chinese-PP. The micro-cycle 1.1 involved two pilot runs. This resulted in a major revamp of the game logic and user interface design. The second micro-cycle, which involved another two pilot runs, prompted us towards some further fine-tuning.

Prior to the first micro-cycle, we designed the game rules with the following principles:

- Based on our previous experiences in the fraction addition games (Boticki et al. 2011), we recommended up to 15 min for, and 15–20 participants to be involved in each game round.
- In preparing each game round, the teacher needed to identify a set of components according to the number of participating students and input them to the system. The choice of components should allow for the construction of as many eligible characters as possible and with at least one global solution (i.e., no student will be left out);
- The game rules, scaffoldings, and incentives should be designed in the way that students are encouraged to strive for balancing the local and global goals.

**Fig. 3:**
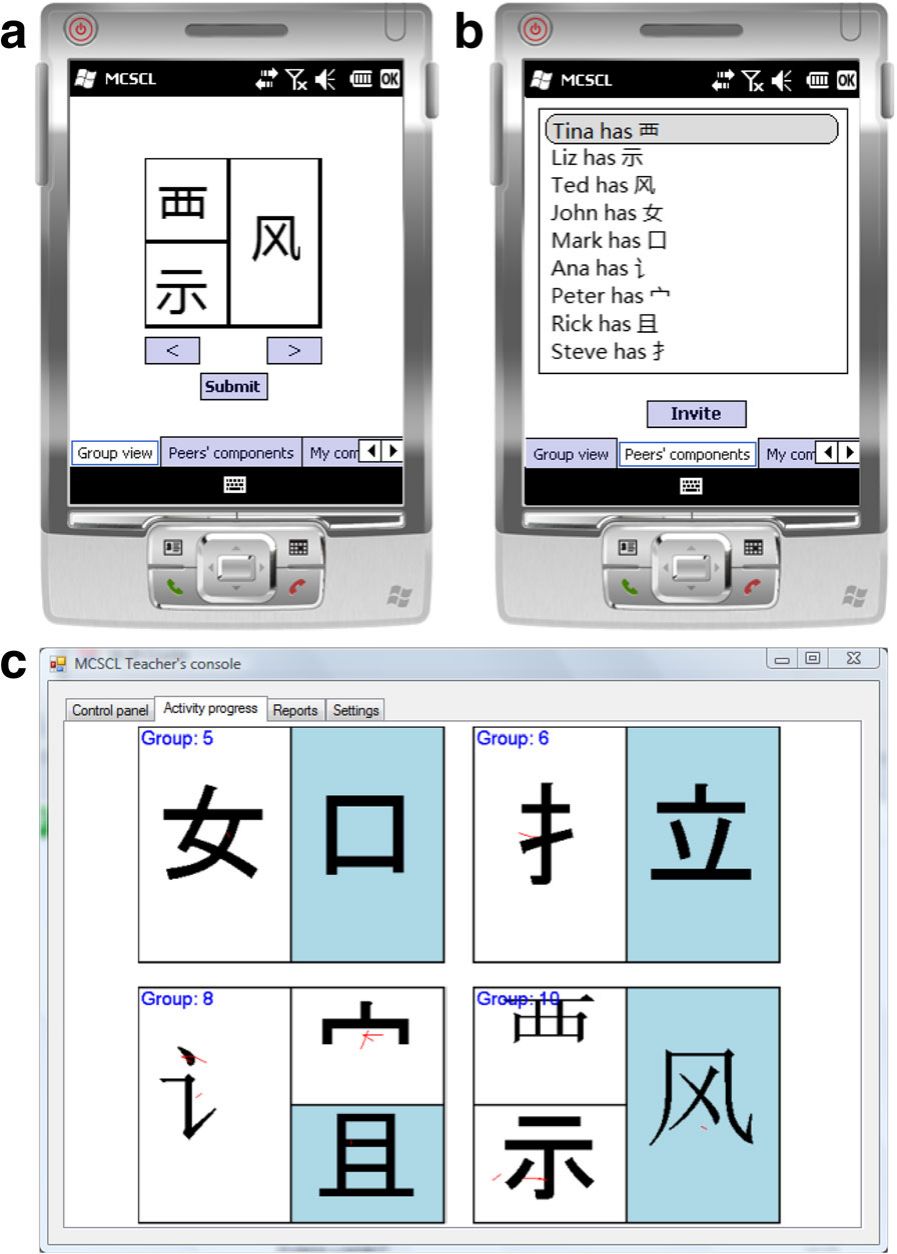
**a** Student’s smartphone application displaying an assembled character. **b** Student’s smartphone application with Chinese language components. **c** Teacher’s view of the Chinese language learning activity. The screen shows the characters from four groups of students each framed into a template

We developed the Chinese-PP Version 1.0 software (Fig. 3). In the beginning of each game round, students choose appropriate components out of the “peers’ components” screen (Fig. 3b), thereby forming a group. Members of each group then discuss and choose one of the general Chinese character configurations to organize their components properly via templates (character configurations) supplied by the Chinese-PP application (arrows < and > in Fig. 3a). The teacher’s console is not only used for controlling the game rounds (e.g., assigning components and starting/terminating a round) but is also projected on a big screen to give students a global view of the game (i.e., showing which characters have already been formed).

In the micro-cycle 1.1, we engaged 37 primary 4 (10 years old) students in the trial runs. We experimented with both the “phone mode” and “card mode.” The card-mode games applied the same game rules as the phone-mode games except without any ICT support, and the students needed to physically cluster together to manipulate their cards in the trial compositions of characters. For the phone-mode games, the students could invite potential group members and accept/reject invitations through the smartphone app. The researchers facilitated all the games by controlling the game pace, provided some hints to the students on possible groupings, verifying students’ groupings, and determining when to terminate a round.

A unique scoring scheme was introduced after the first trial run in order to encourage the balancing of global and local goals. Students earned and accumulated scores by forming legitimate groups: 10 points for a two-component character (same score to be awarded to each group member), 30 points for a three-component character, and 50 points for a four-component character. This was to encourage the students to form bigger groups to fulfill their local goals. This functionality was not automated in Chinese-PP v1.0. In the second pilot run, we computed the “live” scores manually, which was very tedious and time consuming.

All the games were video- and audio-recorded for analysis of students’ gaming and collaborative patterns. The software logs of the students’ interactions during the phone games were also used for triangulation. In addition, a focus group interview was conducted after each trial run to reveal the students’ perceptions in the games and the reasons behind their game-playing behaviors.

The students exhibited similar discussion patterns in all the card and phone game sessions. In each game round, students began with exchanging ideas verbally about arranging the components. Most students first identified a classmate with whom to discuss, and then switched to groups of three to four to discuss alternative possibilities. An initial set of groups was created in the process with a few left-out students still looking for groupings. These left-out students would seek peers’ or researchers’ assistance in identifying other solutions. Some of the students who had already formed groups continued helping other students who do not yet have a group, by offering advices, adding a group member, or even disbanding their own group.

Due to the nature of the domain (Chinese characters) and the UI design of Chinese-PP v1.0, most of the students in the focus groups indicated their preference for playing the card game. They could cluster together and physically manipulate their cards by trial placing them in different spatial configurations. This emergent strategy is what we loosely refer to as “trial-and-error.” During the phone games, the students had to study the “peers’ components” screen (Fig. 3b) and mentally construct characters before deciding whom to invite to form a group. The UI design was seen as imposing additional cognitive burdens on them.

We reflected on the game processes to consider whether we should give up the phone mode and proceed to use the cards for our future study. We let the domain-specific (language acquisition) and cognitive theories inform and guide us in deciding whether we should accommodate or rectify the students’ use of their emergent game strategy. A detailed account of our reflection and improvement process is reported in Wong et al. (2011a)*.* Our decision was to retain and improve the mCSCL solution. Guided by the framework, we revised the learning and technological designs. For example, the students’ trial-and-error strategy in the card mode inspired us to re-design the smartphone UI to show “virtual cards” of individual components that can be dragged and dropped onto the working space to try assembling (*emergent peer scaffolding → technological scaffolding*; left arrow A in Fig. 2). The tedious manual scoring tasks have been automated as well; individual students’ scorings and their overall rankings are dynamically updated in the teacher’s console (*proposed teacher scaffolding → technological scaffolding*; right arrow A in Fig. 2).

**Fig. 4:**
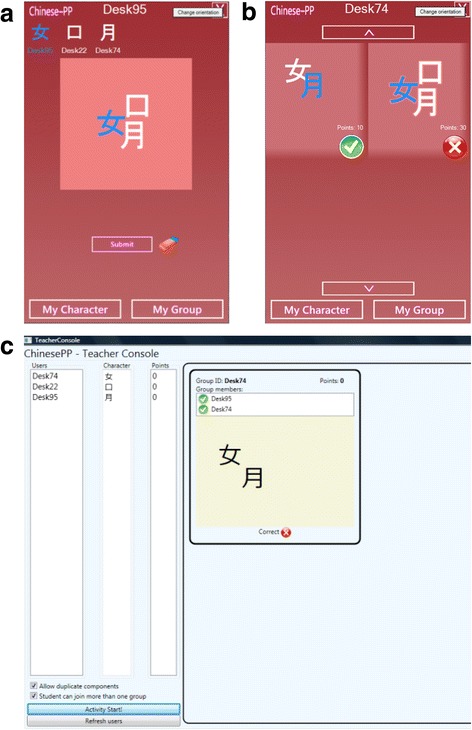
**a** The first client-side application page showing an assembled Chinese character. **b** The second client—side application page showing all assembled groups. **c** Teacher’s application showing all the composed characters for teacher to verify their correctness

We redesigned the UI (Chinese-PP v2.0) to have a client-side layout consisting of the two main application pages (Fig. 4a, b). On the main (first) page, the students are able to see the available Chinese character components that are used to assemble a character (top page menu) and the central assembling canvas. The second main screen displays all formed groups containing a student’s character (specially denoted with the blue color). For each group, the student is able to agree or disagree with the components’ layout.

Figure 4c shows the redesigned teacher application with the user list, Chinese component list, the number of points on the left-hand side, and the assembled group representation area on the right-hand side. Immediately after all students accept a suggested group (green arrow shown on a group representation screen), the teacher’s application allows the teacher to verify the correctness of the assembled character. Only after the teacher has confirmed the correctness of a group’s character are points assigned to its members.

## First cycle, micro-cycle 1.2: more experiments with the revamped game

We advanced to micro-cycle 1.2 by inviting two groups of students to try out the revamped system. The first group was comprised of 15 out of the 37 students who were involved in the experiment in micro-cycle 1.1 (who had moved up to primary 5). The second group was comprised of 16 primary 3 students, also with mixed abilities in Chinese. We repeated the same trial design as the first micro-cycle, except that the researchers no longer facilitated the game sessions. Instead the researchers assisted the Chinese teacher of the future experimental class in our next cycle of study to facilitate the game-related activities. After the experiments, most of the students indicated that they preferred the phone mode. The primary 5 group who used to prefer the card mode in the last micro-cycle told us that the new UI had essentially resolved the “problem” of an inconvenient character composition process, with the additional advantage of letting them see all their peers’ components on one screen—an affordance that the card mode cannot offer.

With the UI revamp, we observed one major difference in the students’ game playing pattern as compared to the first micro-cycle. At the beginning of each round, instead of going straight to peer interactions, they spent more time to drag, drop, and assemble components into characters individually, as though they were attempting a one-player game. This is, in principle, not a drawback since this learning construct allows children to work together and maintains the ability for individual exploration (Inkpen et al. 1995; Wong et al. 2011c; Zurita and Nussbaum 2004). The affordance of the new UI is able to support both private and public cognition.

Our concern was that some of the students just took their time to work individually and did not bother to advance to peer negotiations or group activities. We argue that a “productive” game would consist of two (perhaps intertwining) phases: a personal trial phase, and a grouping and optimization phase. The first phase should not take too long. To manage this potential problem, we advised the teacher to pay attention to the time that the students spent in the personal trial phase of every game round. If the students did not proceed to peer negotiation after 2 min, the teacher reminded the students to move on to checking whether they were invited to join other groups and to begin those discussions. Furthermore, we had to derive more collaborative strategies to train the students and/or formalize good emergent strategies from specific students to train other students, to promote more efficient peer scaffoldings. All of these are reflected in the students’ guidebook (*proposed teacher scaffolding → teacher guidebook & student guidebook*; arrow B in Fig. 2).

Another issue pertained to the teacher’s facilitation of the game sessions. She tended to get too pre-occupied with interacting with the students, answering almost every single student question, and often giving away correct solutions. Occasionally, we needed to remind her to return to the teacher’s console to approve or reject students’ submitted groupings. We related it to our informal observation at a regular Chinese class conducted by her a week before the game sessions and realized that this seemed to be the transference of her usual teaching style to her facilitation of the Chinese-PP sessions. In the future, when such a learning model is translated into a school-based curriculum, individual teachers will be on their own. Our participating teacher’s facilitation style during micro-cycle 1.2 will not work. To identify and address the potential problems, we extracted all the student questions from the transcription of the games and asked the teacher to categorize them and determine suitable strategies to deal with each type of question with the following intentions in mind: (1) to promote student thinking and collaboration, rather than direct instruction; and (2) to reduce her burden in classroom orchestration in the smooth switching between teacher-student interactions and the controlling of the teacher’s console. For example, if a student assembles a character on the phone and asks for the teacher’s verification without consulting the relevant peers, the teacher may advise the student to discuss with those potential group members (*teacher scaffolding → student scaffolding*; arrow C in Fig. 2). All the resulting strategies were documented in teacher’s guidebook for Chinese-PP (*teacher scaffolding → teacher guidebook*; left arrow B in Fig. 2).

## Second cycle: shaping up a curriculum-based intervention and observing students’ growth

The second cycle was a full-fledged pilot study that involved 31 students from a primary 3 class (not the same class who participated in the 1st cycle of study to prevent contamination). The teacher of the experimental class is originated from the People’s Republic of China but went through pre-service teacher education in Singapore and had been teaching in the school for about 5 years. She was involved in a separate mobile-assisted Chinese Language learning project (*<author’s publication>*) for half a year prior to joining the Chinese-PP study. Therefore, she was no stranger to facilitating learning activities with the aid of smartphones.

The entire intervention involved six Chinese-PP learning sessions conducted every fortnight. Specifically, the intervention aimed to progressively establish and enhance the students’ orthographic awareness. Informed by second language acquisition theories and Bloom’s taxonomy, we developed a curriculum framework that outlines major learning goals and activities of the six Chinese-PP sessions. Guided by this framework, the Chinese language teacher of the experimental class designed detailed lesson plans for the individual sessions. The details of our theory-informed curriculum design is reported in (Wong et al. 2013).

Each Chinese-PP session lasted an hour. Originally, we structured each session to consist of 30 min of “pre-game instructions,” and for the students to play the game for 15 min each. The pre-game instructions typically began with the teacher going through a quick review of what had been covered in the previous Chinese-PP session. She then carried out a multimedia-aided presentation to introduce new orthographic knowledge. One example of such knowledge are pictophonetic characters, which refers to characters that are comprised of a component indicating the pronunciation and another representing the semantics. Such instructions were meant to equip the students with the knowledge needed to play the game (with relevant components deliberately selected and assigned to them, such as components that can form pictophonetic characters).

For research purpose, we again collected data through video and audio recordings of all the six sessions, pre- and post-one-to-one semi-structured interviews with six selected students, and system logging. In addition, we conducted paper-based pre- and post-tests, each requiring the students to individually compose as many eligible Chinese characters as possible from 20 given components.

From our reflections on the first three Chinese-PP sessions, we identified a significant drawback. The teacher tended to exceed the planned time limit in delivering the pre-game instructions (up to 40 min). Thus, there was little time left for the students to play the game causing them to rush through the game round or limiting the number of rounds played. The teacher’s focus on delivering pre-crafted instructions can be attributed to typical Singapore teachers’ instructor-centric belief (Hogan and Gopinathan 2008; Lim and Chai 2008) that students must be thoroughly taught certain concepts or knowledge before they can proceed to apply those in hands-on activities.

Thus, working together with the teacher, we revised the standard structure of individual Chinese-PP sessions. We persuaded the teacher that “learning by doing” (Anzai and Simon 1979) is more effective than didactic instruction. The last three Chinese-PP sessions were re-structured into three stages: (1) “pre-game warm-up” (15 min) that included a quick introduction to new orthographic rules with more examples and execution of simple small-group paper-and-pen-based activities; (2) game playing (20 min per batch); and (3) “post-game recalling” (5 min) where students were asked to recall and relate the characters they had composed during the game to the orthographic rules that they learned in the present and past warm-up stages.

With the restructuring, the teacher became more conscious of time management and in explicitly relating students’ composed characters during the games with the orthographic rules. The changes were documented (*proposed and emergent teacher scaffolding → teacher guidebook*; left arrow B in Fig. 2). An encouraging development is that some students started to explicitly refer to the orthographic rules during peer negotiations, thus reinforcing their understanding in the domain knowledge.

With further real-time observation and after-game analysis of the students’ intra-group and inter-group interactions, we discovered even more emergent domain-specific strategies used by the students to figure out more alternative characters. Six of such strategies was identified, as shown in Table 1. They were not taught these strategies but these were clearly derived from and rooted in the orthographic rules that the teacher had introduced to them. These strategies were also documented; and future teachers may consider explicitly recommending such strategies to weaker students (*emergent student scaffolding → teacher guidebook & student guidebook*; arrow B in Fig. 2).

**Table 1:**
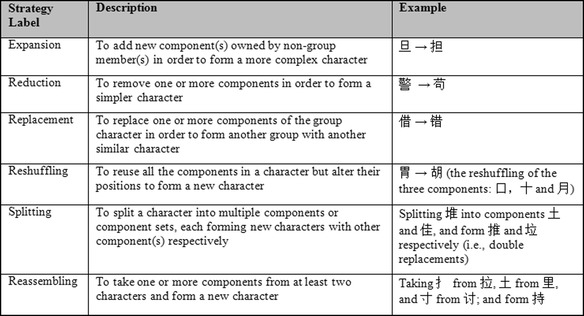
Student strategies for figuring out alternative characters during the Chinese-PP games

We compared the participating students’ pre- and post-test scores (in terms of numbers of eligible characters being identified) by conducting a paired sample *t* test and yielded *t* = − 4.377, *p* < .05 (see Table 2). This indicates a significant improvement in individual students’ capability of composing alternative characters from the given components (e.g., merging口and木to become杏, or alternatively, 呆; both are legitimate Chinese characters).

**Table 2:** Result of pair sample *t* test on pre- and post-test scores of the students involved in second cycle intervention (*n* = 31)

	Pre-test	Post-test
Mean	10.48387	13.90323
Variance	16.1914	29.49032
Observations	31	31
Pearson correlation	0.612393	
Hypothesized mean difference	0	
df	30	
*t* Stat	− 4.37708	
P(T ≤ *t*) one-tail	6.71E−05	
t Critical one-tail	1.697261	
P(T ≤ *t*) two-tail	0.000134	
t Critical two-tail	2.042272	

## Discussion and conclusion

We have described Chinese-PP, a game-based learning approach to address the L2 learners’ need to enhance their understanding of the structure of Chinese characters. Such a learning design provides a novel yet linguistically sound way of learning Chinese characters which is very different from the conventional instructional styles used by teachers. We worked out complex game rules to meet the learning goals based on theories of language acquisition and collaborative learning. However, the design and the use of this approach and technology may pose serious classroom orchestration challenges for teachers should it be translated to regular classroom modules. Individual teachers’ diversified instructional or interactional styles may play a crucial part in facilitating the collaborative learning processes in Chinese-PP.

With the eventual aim of translating the learning model to curricular practices in the school, we adopted DBR and in turn derived the distributed scaffolding design framework to help us in systematically codifying and improving/transforming various types of proposed or emergent scaffoldings, and consequently *redefining/redistributing* the roles of the teachers, the students, and the technology. From a classroom orchestration perspective, the design framework identifies and recognizes the real challenges faced by the teacher and the students in using the collaborative technology in the classroom. From a design perspective, the framework, through a user-centered design approach, informs the next iterative design of the provision of scaffolds based on analysis of the emergent usage of the current design by students and the teacher.

Many researchers of learning technology tend to conduct their studies in the way of pooling resources (e.g., more manpower, technological tools, and server bandwidths) and having themselves (the researchers) playing a dominant role in learning designs and enactments. Conversely, teachers are virtually transformed into assistants or facilitators by the side (Wong et al. 2010). When individual teachers facilitate such classes on their own, they usually have fewer resources, such as no dedicated teaching or technical assistants, at their disposal. In our study, we attempted to address this issue by using our distributed scaffolding design framework to modify critical variables in the success of this approach and gradually bring the Chinese-PP model closer to being adopted into a regular curriculum.
